# The Association of Lung Cancer and Sarcoidosis: A Systematic Review

**DOI:** 10.7759/cureus.21169

**Published:** 2022-01-12

**Authors:** Mirra Srinivasan, Santhosh Raja Thangaraj, Hadia Arzoun, Lekshmana Bharathi Govindasamy Kulandaisamy, Lubna Mohammed

**Affiliations:** 1 Internal Medicine, California Institute of Behavioral Neurosciences & Psychology, Fairfield, USA; 2 Pathology, California Institute of Behavioral Neurosciences & Psychology, Fairfield, USA

**Keywords:** pulmonary nodules, granulomas, incidence, sarcoidosis, neoplasm, lung cancer

## Abstract

Lung cancer has been the leading cause of cancer-associated deaths worldwide. While numerous reasons, including tobacco smoking, may lead to lung cancer, the purpose of this article was to explore the association between sarcoidosis, a multisystem granulomatous disorder, and lung neoplasms. A literature search was done on multiple databases with appropriate keywords, and the authors selected case reports where patients were diagnosed with sarcoidosis and lung cancer. These reports were analyzed in detail, and nine reports were included in this study. Each case was evaluated for the presenting symptoms, age, gender, and diagnostic procedures, including a follow-up analysis. After the evaluation, it can be concluded that sarcoidosis and lung cancer can occur simultaneously, despite being rare. Appropriate diagnostic procedures, including histopathological examination of the affected lymph nodes, showed either cancerous or non-cancerous cells (granulomas), thus altering the treatment on a case-by-case basis. Being aware of all possible associations between these two diseases could alter the clinical management, whether curative or palliative, and clinicians must rule out metastatic cancer in individuals with sarcoidosis-like clinical and radiographic features.

## Introduction and background

In the present decade, lung cancer seems to be the number one cause of cancer-related deaths in both females and males across the globe, merely due to an insidious onset, with increased metastasis and poor prognosis [1]. Although the primary cause of lung cancer is tobacco, other potential risk factors include environmental exposure to cigarette smoke, radon, occupational carcinogens, and pre-existing nonmalignant lung illness, in addition to genetic variables, which have a role in changing an individual's risk of lung cancer, according to recent studies in molecular biology [2]. Cancer of the lung can be classified into two types according to the histological pattern, namely, small-cell lung carcinomas (SCLC) and non-small cell lung carcinomas (NSCLC), where the prognosis of each type will vary [3].

**Figure 1:**
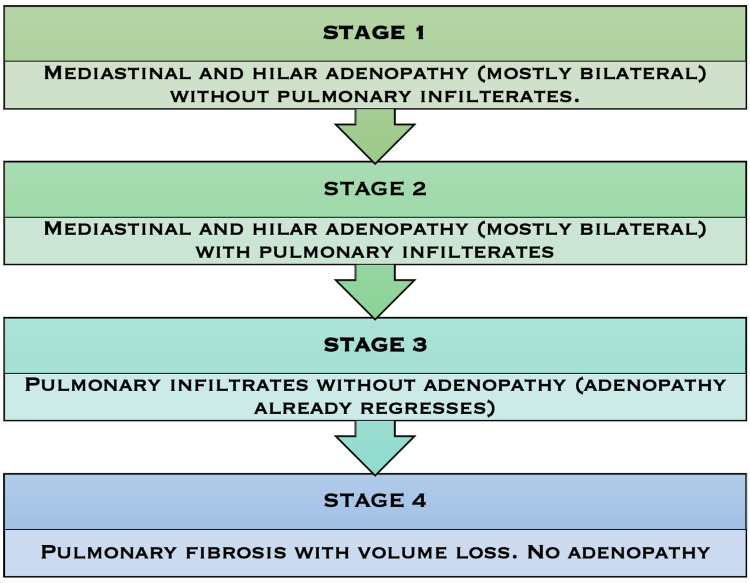
The four stages of pulmonary sarcoidosis according to the histopathological classification. Figure created by the author on Microsoft PowerPoint (Microsoft Corporation, Redmond, WA).

Sarcoidosis is a systemic illness with an unknown cause that manifests as non-caseating granulomas in any organ, most often the lungs and intrathoracic lymph nodes [4]. Despite the fact that the etiology of sarcoidosis is unclear, it is assumed to be the result of an excessive immune response to some environmental triggers in genetically susceptible patients [5]. Several studies have reported the prevalence of sarcoidosis in previously unstudied groups, demonstrating that sarcoidosis is on the verge of increasing globally. According to one study, the incidence of sarcoidosis in the United States ranged from 7.6 to 8.8 per 100,000 population per year [6]. At presentation, the most common clinical symptoms include cough, dyspnea, bronchial hyperreactivity, fatigue, night sweats, weight loss, and erythema nodosum; however, on the other hand, up to 50% of the patients are asymptomatic, with abnormalities identified incidentally during chest radiography [7]. There is no one definitive diagnostic test [4], other than the exclusion of other causes of granulomatous inflammation based on histopathologic examination and clinical presentation [8]; hence, early and accurate diagnosis of this disease is challenging. Although the best treatment for sarcoidosis is unknown, corticosteroid medication has long been the standard of care for individuals with extreme symptoms and progressing pulmonary disease or substantial extrapulmonary illness. Immunosuppressive treatment may be required in refractory or complicated instances. Even though the clinical course is chronic or progressive in 10% to 30%, spontaneous remission has been documented in around two-thirds of patients [9]. Despite vigorous therapy, some individuals with severe, progressing illnesses may suffer life-threatening respiratory, cardiac, or neurologic problems [4]. Lungs being the most commonly affected organ due to sarcoidosis and pulmonary sarcoidosis can be classified into four stages, as shown in Figure 1 below [8].

This research aims to examine the relationship between sarcoidosis and lung cancer and determine whether there is a correlation between the two by emphasizing uncommon instances reported globally in the last 10 years and providing insight into how physicians can effectively handle such rare encounters.

Methodology

The following databases were explored, namely, PubMed, Google Scholar, Science Direct, and Directory of Open Access Journals (DOAJ), and all relevant reports were retrieved electronically without the use of automated tools upon entering the keywords mentioned below. The Boolean scheme was employed to the keywords while also incorporating the Medical Subject Heading (MeSH) strategy. The articles retrieved were checked for titles/abstracts while setting an inclusion/exclusion criterion, given below. The Preferred Reporting Items for Systematic Reviews and Meta-Analyses (PRISMA) guidelines 2020 were adhered to in this review article [10].

Inclusion and exclusion criteria

The authors included only case report studies from the past 10 years (2011-2021) in the English language with open access full-text reports across the globe. All other study designs were excluded, including studies prior to 2011, non-English, and non-full text articles. All the included studies fulfilled the quality assessment.

Keywords

MeSH keywords searched in PubMed included: lung cancer OR ((“lung neoplasms/analysis"[Mesh] OR "lung neoplasms/diagnostic imaging"[Mesh] OR "lung neoplasms/etiology"[Mesh] OR "lung neoplasms/immunology"[Mesh] OR "lung neoplasms/pathology"[Mesh] )) AND sarcoidosis OR sarcoid OR Besnier-Boeck-Schaumann disease OR ("sarcoidosis/analysis"[Mesh] OR "sarcoidosis/complications"[Mesh] OR "sarcoidosis/diagnosis"[Mesh] OR "sarcoidosis/immunology"[Mesh] OR "sarcoidosis/pathology"[Mesh]).

Keywords searched in other databases included: lung cancer, neoplasm, sarcoidosis, incidence, granulomas, and pulmonary nodules.

Quality assessment

**Figure 2:**
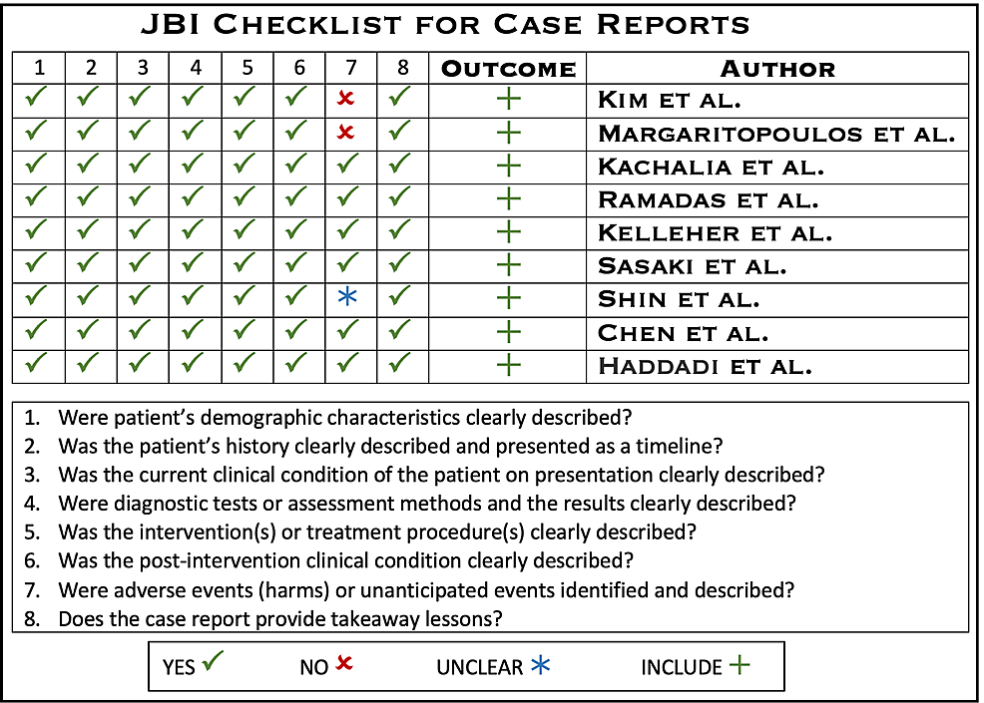
Quality assessment of the included reports. Figure created by the author on Microsoft PowerPoint.

During the selection process, the study team employed an appropriate quality appraisal tool, the Joanna Briggs Institute (JBI) Critical Appraisal Checklist for Case Reports, and two researchers worked independently on data selection and extraction. In situations where the researchers could not agree, they discussed the study designs, inclusion and exclusion criteria, intervention employed, and results measured. In instances of a dilemma, a third reviewer was approached to help resolve disagreements and find common ground. The quality of the selected case reports is depicted in Figure 2 below.

Results

**Figure 3:**
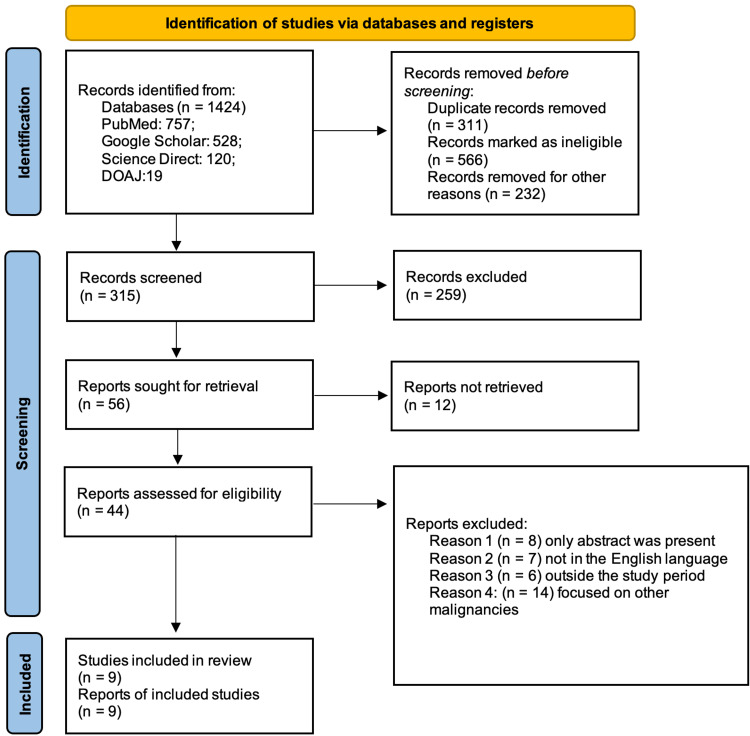
Search results depicted in the PRISMA flow chart 2020. PRISMA, Preferred Reporting Items for Systematic Reviews and Meta-Analyses; DOAJ, Directory of Open Access Journals.

The search strategy used in this study included the above-mentioned databases and yielded 1,424 articles, out of which 311 were removed as they were duplicates using EndNote (Clarivate, London, UK), 566 were removed due to ineligible records on the basis of abstract/title and 232 were removed due to other reasons such as irrelevant study design and poor quality. A total of 315 records were screened, out of which 259 were excluded based on the relevance and the inclusion/exclusion criteria. Twelve reports could not be retrieved, and the final screening was down to 44 reports to check for quality and eligibility. Finally, nine studies were included for this review. The PRISMA flow diagram is depicted in Figure 3 below [10].

The patient demographics of the included case reports are described in Table 1 below [9,11-18].

**Table 1:** Summary of patient characteristics of the included reports.

Author	Year	Patient details (age, sex, and ethnicity)	Symptoms at presentation	Duration of symptoms
Kim et al. [9]	2011	62/female/Asian	Chronic cough	Four years
Margaritopoulos et al. [11]	2012	65/female/Caucasian	Progressive breathlessness, dry cough, fatigue, arthralgias, and mild weight loss	Ten months
Kachalia et al. [12]	2014	80/Female/African American	Cough, chest pain, asymptomatic anemia, and an unintentional 21-pound weight loss	Six months
Ramadas et al. [13]	2016	65/female/African American	Right-sided shoulder pain and exertional dyspnea	Two months
Kelleher et al. [14]	2018	44/female/African American	Dyspnea, night sweats, and unintentional 15-pound weight loss	Two months
Sasaki et al. [15]	2018	69/female/Asian	Asymptomatic	Not applicable
Shin et al. [16]	2020	65/male/Asian	Asymptomatic	Not applicable
Chen et al. [17]	2021	50/male/Asian	Chest tightness and productive cough	Two weeks
Haddadi et al. [18]	2021	71/male/not mentioned	Exertional dyspnea	One and off x two years

The background and highlights of the included case reports are summarized in Table 2 below [9,11-18].

**Table 2:** Highlights of the included reports. AFB: acid-fast bacilli; CXR: chest X-ray; TTF1: thyroid transcription factor 1; VATS: video-assisted thoracic surgery; EGFR: epidermal growth factor receptor; ALK: anaplastic lymphoma kinase; CT: computed tomography; FDG-PET: fluorodeoxyglucose-positron emission tomography; FDG: fluorodeoxyglucose; EBUS: endobronchial ultrasound; CD56: neural cell adhesion molecule; SCLC: small cell lung cancer; TBNA: transbronchial needle aspiration; BHL: bilateral hilar lymphadenopathy; EML4-ALK: echinoderm microtubule-associated protein-like 4 gene- anaplastic lymphoma kinase; NSCLC: non-small-lung cancer; ACE: angiotensin-converting enzyme; COPD: chronic obstructive lung disease; GATA3: GATA binding protein 3; p63: transformation-related protein 63; CK7: cytokeratin 7.

Author	Highlights of the Included Reports
Kim et al. [9]	According to the pathology results, the tumor was a well-differentiated adenocarcinoma, and all of the enlarged mediastinal lymph nodes were granulomas without cancer metastasis. There was no sarcoidosis in the lung mass and no scar lesion, despite the fact that sarcoidosis and lung cancer occurred simultaneously, and the prognosis was said to be unaffected by sarcoidosis. This case of sarcoidosis and lung cancer that happened at the same time was effectively treated.
Margaritopoulos et al. [11]	Non-caseating granulomas were identified in this patient through endobronchial biopsies. While non-caseating granulomas can be seen near tumors, the patient had a video-assisted thoracoscopy and an axillary lymph node resected, both of which confirmed the presence of non-caseating granulomas and the diagnosis of sarcoidosis. Steroids were administered, after which the clinical course improved. Sarcoidosis can resemble infectious, malignant, and granulomatous disorders, increasing the risk of lung cancer in individuals. Sarcoidosis should always be considered in the differential diagnosis of individuals with a lung mass encasing and narrowing bronchial and vascular systems, as well as a pericardial effusion.
Kachalia et al. [12]	Multiple developed homogeneous non-caseating granulomatous inflammation was seen in biopsies from both the right upper lobe lesion and a mediastinoscopic-derived lymph node. However, stains and cultures for AFB/fungal organisms were negative. The patient's condition improved after taking oral steroids. She returned six months later with increased dyspnea, and a CXR revealed bilateral pleural effusions. TTF1 positive adenocarcinoma cells were discovered during thoracocentesis, and VATS revealed multiple pleural, pericardial, and diaphragmatic metastases. TTF1 adenocarcinoma and EGFR mutation were also found in the biopsy; however, ALK was not evident. While proof of an association between sarcoidosis and lung adenocarcinoma is still lacking, practitioners should rule out metastatic cancer in patients with clinical and radiographic symptoms compatible with sarcoidosis.
Ramadas et al. [13]	A CT scan of the thorax revealed a mass in the right upper lobe, as well as mediastinal and bilateral hilar lymphadenopathy. The right upper lobe and the mediastinal and hilar lymph nodes exhibited enhanced uptake on an FDG-PET scan. Non-necrotizing granulomas with no signs of malignancy were discovered during an EBUS biopsy of numerous lymph nodes. The histology of the right upper lobe lesion was compatible with small cell carcinoma after a CT-guided biopsy. Sarcoidosis and small cell cancer were suspected as causes of the enlarged and aggressive mediastinal and hilar lymph nodes. After two cycles of chemotherapy (carboplatin and etoposide) and radiation treatment, a follow-up CT thorax revealed a reduction in the nodule's size and mediastinal and hilar lymph nodes. In this patient, it was impossible to tell whether the nodal involvement was due to sarcoidosis, small cell cancer metastases, or a sarcoid-like response; however, given the longstanding history of sarcoidosis that was treated multiple times could have predisposed to the lung neoplasm.
Kelleher et al. [14]	After taking prednisone for a presumed asthma exacerbation, the patient developed worsening dyspnea, night sweats, and unintentional weight loss. A sizable left lower lobe mass and hilar lymphadenopathy were discovered; furthermore, upon CT-guided biopsy, the lung mass was found to be a multifocal non-necrotizing granuloma with multinucleated giant cells. Although this observation is consistent with sarcoidosis, it might also be a sarcoid-like response caused by a hidden tumor. A more thorough bronchoscopy and mediastinoscopy biopsy revealed granulomatous inflammation with no signs of cancer or infection. A strong index of suspicion is required for rapid diagnosis and appropriate care when a malignancy is suspected.
Sasaki et al. [15]	A CT of the chest revealed a mass lesion in the lower part of her left main bronchus four years after the patient was diagnosed with sarcoidosis, and an FDG-PET scan revealed intense FDG activity in the left lower lobe bronchus, the lymph node just above the cardia, and the right side of the upper abdominal aorta. The proliferation of small cells with a high nucleus-cytoplasm ratio positive for CD56 and synaptophysin was seen on histological examination of the left main bronchus. In a stage of spontaneous remission from sarcoidosis, the patient was diagnosed with extensive-stage SCLC with abdominal lymph node metastases. After four chemotherapy sessions (cisplatin and etoposide), the tumor surrounding the bronchus of the left lower lobe and lymph node metastases were drastically decreased and showed almost a complete response. Before and after the chemotherapeutic therapy, the pulmonary sarcoidosis was identical. Even though sarcoidosis is in remission, it is essential to monitor the possibility of carcinogenesis, especially in smokers.
Shin et al. [16]	Sarcoidosis was diagnosed based on the pathology results through the EBUS-guided TBNA with sufficient core samples from subcarinal and bilateral interlobar lymph nodes. Following five months of oral corticosteroid medication, a follow-up chest CT revealed a newly acquired right lower paratracheal lymphadenopathy as well as deteriorating right hilar lymphadenopathy. A repeat bronchoscopy and an EBUS-guided biopsy of the right upper lobe and right lower paratracheal lymph revealed adenocarcinoma of the lung. If a patient with sarcoidosis does not respond to early corticosteroid therapy, the practitioner should consider lung cancer as a possibility.
Chen et al. [17]	EML4-ALK-positive NSCLC with accompanying non-caseating granuloma was discovered during a CT-guided biopsy of a lesion in the left superior lobe. Crizotinib was used to treat this patient. A chest computed tomography scan one month later indicated a significant reduction in the size of the left upper lobe nodule, but the lesions in the right lung had progressed. Granulomas were seen in the right supraclavicular lymph nodes, but no tumor cells were found in the samples. The level of the ACE was abnormally high. All lesions showed a substantial response after one week of methylprednisolone therapy. Non-caseating granulomas were seen in both lung tissues and lymph nodes after extensive resection of the lung carcinoma, leading to a diagnosis of EML4-ALK-positive NSCLC with sarcoidosis, which is more prevalent in younger individuals, but there are no other shared risk factors.
Haddadi et al. [18]	An EBUS scan revealed non-necrotizing granuloma in a COPD patient, who was reviewed for sarcoidosis treatment. A nodule biopsy was also performed on the patient to rule out cancer sarcoid syndrome. A poorly differentiated adenocarcinoma of the lung was reported that was positive for GATA3, P63, CK7, and TTF-1. The patient underwent surgical intervention, and no signs or symptoms of systemic sarcoidosis were documented. According to the study, identifying cancer earlier improves the patient's outcome and prognosis.

## Review

This section discusses the relationship between the incidence of lung cancer in patients diagnosed with sarcoidosis and thus explores various case reports on this topic. The authors have also documented the limitations of this review.

According to epidemiological research, sarcoidosis and neoplasms may be etiologically associated in at least 25% of patients where both are present [12]. The association between sarcoidosis and lung cancer has been studied in several studies, one of which is the study by Brincker and Wilbek, which looked at 2,544 patients and found that lung cancer was three times more common in those with sarcoidosis [9]; however, the exact relationship between sarcoidosis and lung cancer remains unknown [15]. The reasons for this association were postulated in 1963 by the Sakula study, where three possibilities were pointed out: (1) sarcoidosis precedes lung cancer and is somehow connected to, and may even potentially trigger, the malignant transformation due to the postinflammatory scar tissue; (2) sarcoidosis may develop as a consequence of lung cancer; and (3) the onset of sarcoidosis precedes the onset of lung cancer, and the incidence of lung cancer and sarcoidosis is absolutely coincidental [19]. Other theories have been recently postulated where sarcoidosis-induced cell-mediated immunological abnormalities have a role in lung cancer development; another theory proposes sarcoidosis as an immune response to tumor antigens. This theory is based on findings of solid and lymphohematogenous tumors generating a systemic granulomatous response similar to sarcoidosis [12]. Research conducted in Japan from 1980 to 2007 detected the first case with sarcoidosis to have lung cancer 16 years following the diagnosis of sarcoidosis; the second case developed two separate metachronous lung malignancies 18 and 10 years later. The third case identified both disorders at the same time [20]. A non-caseating granuloma can help confirm a diagnosis of sarcoidosis in optimal clinical settings [11]; furthermore, the existence of non-caseating granulomas in correlation with a malignancy, referred to as a sarcoid-like response, has been observed to exist either in proximity to cancer's location or around the neighboring lymph nodes [14]. Because non-caseating granulomas have been observed in some instances of small cell lung cancer, the presence of granulomas does not always rule out malignancy [14], in addition, both sarcoidosis and lung cancer have mediastinal lymph node involvement [14]. Sarcoid-like responses can occur in both hematologic and solid tumors [5]; moreover, one research found a connection between sarcoid-like reactions and lung cancer, which are histologically indistinguishable from granulomas seen in typical sarcoidosis, which was thought to result from an immune response to local tumor products [14]. As a result, "true" sarcoidosis and local sarcoid-like responses in cancer patients may be misinterpreted. There is also additional evidence that finding sarcoid responses in non-small cell lung cancer patients' regional lymph nodes predicts a decreased likelihood of disease recurrence following surgical resection [13]. The sarcoid response of lymph nodes in individuals with non-small cell lung carcinoma is only evident in stage I of lung cancer, according to the Steinfort study, and patients with cancer have had a better result, linked to a significant cell-mediated antitumor response [18]. It is difficult to predict whether noncaseating epithelioid cell granulomas coexisting with lung cancer are sarcoid response or confirmed systemic sarcoidosis in cases where both are discovered simultaneously [20]. Although sarcoidosis and lung cancer coexistence are exceedingly rare, with less than 1% frequency, it is most commonly encountered in squamous cell lung malignancies [13] and has been documented to be associated with a lower survival rate [21]. Hence, lung cancer, a condition seen in sarcoidosis patients, should be included in the differential diagnosis when concerning symptoms are identified, whether due to causality or coincidence [20]. In a patient with sarcoidosis, performing a lung biopsy from any nodular lesion is critical for differential diagnosis and early treatment interventions [18].

When pulmonary sarcoidosis and primary or secondary lung tumors coexist, it might be challenging to diagnose, thus leading to an obscured preoperative staging [22]. Computed tomography (CT) scanning is a good predictor of mediastinal staging in lung cancer, but it cannot determine the nature of lymphadenopathy or a contralateral radiographic anomaly in the presence of sarcoidosis. Due to the semiquantitative measurement of differential standardized uptake value (SUV) of fluorodeoxyglucose (FDG), positron emission tomography (PET) scanning can distinguish between benign and malignant lung lesions, further aiding with preoperative staging by determining the nodal status [22]. In certain cases, lung cancer may be diagnosed first, and because of the multiple lymphadenopathies, excision of the tumor may not be considered if sarcoidosis is not confirmed. As a result, it is critical to evaluate the coexistence of these two disorders and distinguish non-caseating granulomas-induced lymphadenopathies from metastasis [18]. In about 3% to 5% of NSCLC patients, rearrangements of the anaplastic lymphoma kinase (ALK) gene (ALK-positive) could render as an oncogenic trigger. Sarcoidosis and ALK-positive NSCLC are uncommon together, and ALK-positive lung cancer is likely to spread rapidly. As a result, a co-occurrence of sarcoidosis and metastatic lung cancer is more readily misinterpreted by radiological testing [17].

Another viewpoint of this review is to emphasize the safety margin of immunotherapy where alternate theories state that sarcoidosis may be caused by lung cancer or therapeutic interventions including chemotherapy, tyrosine kinase inhibitors, or immunological checkpoint inhibitors [17] such as ipilimumab and nivolumab [15], which cause local sarcoid responses [15,17]. The primary lung cancer lesion in Chen et al.'s study was in the left upper lobe; however, granulomas were discovered in the nonneoplastic right lung and supraclavicular lymph node. Furthermore, the ACE level in the blood was abnormally high. These data show that the granulomas were caused by true sarcoidosis rather than sarcoid responses in response to lung malignancy [17]. Only two reports of pulmonary sarcoidosis in the setting of immunotherapy for lung cancer treatment have been published, both of which reported cutaneous sarcoidosis rather than pulmonary sarcoidosis; however, Fakhri et al. demonstrated pulmonary sarcoidosis in a patient with NSCLC just four months after starting chemotherapy (carboplatin/pemetrexed) plus pembrolizumab combination therapy suggesting that immune checkpoint inhibitors could cause immuno-induced sarcoidosis, which may be misinterpreted as the progression of lung cancer. Hence, it is critical to confirm the diagnosis through tissue biopsy [23]. Some other studies have documented sarcoid-like responses arising due to microbial infections, including leishmaniasis, tuberculosis, and coccidioidomycosis; hence, biopsies from two nonadjacent locations are advised in patients of atypical sarcoidosis to rule out an active tumor or infection [14].

One study documented pleural effusion as an uncommon manifestation of sarcoidosis relapse with less than 10% estimated incidence and is more likely in individuals with the active parenchymal disease. Increased capillary permeability with limited pleural space inflammation may result from pleural involvement. In recent research of sarcoidosis patients, chest ultrasonography was used to diagnose pleural effusion in five out of 181 cases, whereas biopsy-proven diagnosis was established in only two cases. The effusions are lymphocytic exudates that are usually small to moderate with a high CD4/CD8 ratio of T lymphocytes. It is critical to rule out infections or malignancies as probable etiology with the assistance of a pleural biopsy [11].

The evidence on the association between sarcoidosis and lung cancer has also had opposing views, where some studies imply that individuals with sarcoidosis have a greater risk of lung cancer and mortality from lung cancer, others demonstrate that the incidence is not higher than predicted. However, the ongoing inflammatory process and scarring associated with sarcoidosis, along with anomalies in cell-mediated immunity, might be a potential trigger for carcinogenesis [13]. Sarcoidosis contains tumor-promoting and immune-regulatory cell subsets, which have the ability to induce metastasis; in particular, myeloid-derived suppressor cells (MDSC) may play a vital role in metastatic transformation. A sarcoidosis-related inflammation is caused by the expression of interferon-γ (IFN-γ), interleukin-6, interleukin-23, and S100A8/A9, in turn leading to the immunosuppressive activity of MDSC. S100A8 and S100A9, myeloid-related proteins that act as growth factors, were found to be elevated in patients diagnosed with sarcoidosis along with an enhanced cytoplasmic expression in monocytes multinucleated giant cells in granulomas. S100A9-expressing MDSC were also discovered as essential contributors in facilitating tumor dissemination in a melanoma and lung carcinoma mouse model and are regarded as the most aggressive tumor promoters [21].

Limitations

This review mainly focuses on case reports, and other types of study designs were not included for the sole reason of analyzing the association of lung cancer and sarcoidosis extensively on a case-by-case basis. The intention for this review was to aid healthcare practitioners to be aware of the rare connection between these two illnesses and to monitor the overall health of these patients with a multidisciplinary approach at periodic intervals. Another limitation of the review is that the authors did not get into the details about the other multisystem symptoms or complications of sarcoidosis to stay focused on the research question; however, the multisystem approach to sarcoidosis management is a separate domain and could be a research topic in and of itself.

## Conclusions

Sarcoidosis may mimic infectious, neoplastic, and granulomatous diseases, as well as its potential to predispose people to lung cancer. The significance of keeping a diversified differential diagnosis and the need for a detailed assessment is essential. Although sarcoidosis and lung cancer rarely coexist, physicians should thoroughly investigate the potential of lung cancer in sarcoidosis patients who do not respond to early corticosteroid treatment in addition to obtaining a biopsy to establish pathological findings from any suspected lung lesion. While evidence of an association between sarcoidosis and lung adenocarcinoma is still lacking, the clinicians need to make sure that no lesion should be overlooked in these individuals without a biopsy if sarcoidosis is suspected. This structured approach can enhance the patient's outcome and prognosis while also detecting malignancy at an earlier stage. Atypical pulmonary sarcoidosis symptoms are difficult to diagnose since the disease's clinical and radiological signs are similar to those of a malignancy. Given the diagnostic conundrum of not being able to differentiate sarcoidosis and lung cancer, it is better to treat the nodal involvement as small cell lung cancer metastasis because failure to do so might result in relapse and jeopardize survival. In conclusion, sarcoidosis should always be considered in the differential diagnosis of individuals who appear with a lung mass. Case reports such as these, if encountered, need to be reported, especially given the rare association between lung cancer and sarcoidosis, and additional data will add value to the existing literature, allowing for more in-depth analysis and possibly finding concrete answers to the association between lung cancer and sarcoidosis.
